# The Anti-colitis Effect of *Schisandra chinensis* Polysaccharide Is Associated With the Regulation of the Composition and Metabolism of Gut Microbiota

**DOI:** 10.3389/fcimb.2020.519479

**Published:** 2020-10-21

**Authors:** Lianlin Su, Chunqin Mao, Xiachang Wang, Lin Li, Huangjin Tong, Jing Mao, De Ji, Tulin Lu, Min Hao, Ziyan Huang, Chenghao Fei, Kewei Zhang, Guojun Yan

**Affiliations:** ^1^College of Pharmacy, Nanjing University of Chinese Medicine, Nanjing, China; ^2^The Key Laboratory of Chinese Herbal Medicine Processing of Jiangsu Province, Nanjing, China; ^3^Affiliated Hospital of Integrated Traditional Chinese and Western Medicine, Nanjing University of Chinese Medicine, Nanjing, China; ^4^Jiangsu Province Academy of Traditional Chinese Medicine, Nanjing, China; ^5^School of Medicine and Life Sciences, Nanjing University of Chinese Medicine, Nanjing, China

**Keywords:** *Schisandra chinensis*, polysaccharide, ulcerative colitis, gut microbiota, short chain fatty acid, inflammation

## Abstract

**Background:** The pathogenesis of inflammatory bowel disease (IBD) is linked to an intricate association of environmental, microbial, and host-related factors. Polysaccharide affects host immunity by regulating the composition and metabolism of gut microbiota is the common mechanism of disease resistance. However, the efficacy and mechanism of *Schisandra chinensis* polysaccharide (SCP) in the treatment of inflammatory bowel disease have not been studied.

**Objective:** To explore the effect and mechanism of SCP on dextran sodium sulfate (DSS) - induced ulcerative colitis (UC) in mice.

**Materials/Methods:** In this study, we established a mouse model of UC, and used SCP for treatment intervention. The biochemical indexes related to inflammation were determined by ELISA kit, and the therapeutic effect of SCP on UC was clarified. Then, 16S rDNA sequencing was used to study the effect of SCP on the composition and diversity of gut microbiota. At the same time, GC-MS was used to determine the content of short chain fatty acids in intestinal contents. Finally, the relationship among gut microbiota, short chain fatty acids and inflammatory factors was analyzed, and to comprehensively explain the effect and mechanism of SCP on UC.

**Results:** The results showed that SCP could significantly improve the physiological state of UC mice and regulate the level of inflammatory factors to normal levels. Meanwhile, SCP could significantly regulate the imbalance of gut microbiota and increase the content of SCFAs. In addition, the results of the correlation between gut microbiota and SCFAs showed that butyric acid, isobutyric acid and valeric acid had the highest correlation with gut microbiota.

**Conclusion:** In conclusion, this research showed that SCP can inhibit inflammatory bowel disease by regulating the composition and metabolism of gut microbiota, and indicating that SCP may be used as adjuvant therapy for IBD patients.

## Introduction

Inflammatory bowel disease (IBD) mainly includes Ulcerative colitis (UC) and Crohn's disease (CD). It is a chronic recurrent disease characterized by intestinal inflammation and epithelial injury. It is difficult to cure and poses a serious threat to human health (Van Der Veen et al., 2009). In recent years, the incidence of IBD has been increasing year by year and has gradually evolved into a global disease. In addition, epidemiological data show that the duration and severity of chronic colitis is an important risk factor for colon cancer associated with colitis (Torres et al., 2013). The main pathological mechanism of IBD is not clear yet. There are many causes of IBD, such as genetic factors and environmental factors, which may lead to innate and adaptive mucosal immune responses. In recent years, intestinal flora imbalance has been recognized as the main pathological mechanism of IBD (Swidsinski et al., 2008).

More and more studies have shown that dietary fiber (DF), such as polysaccharides, can affect the composition of gut microbiota (Zhang et al., 2017; Cai et al., 2019). Dietary fiber provides an important energy source for the bacterial activity of intestinal microflora and can directly or indirectly affect the intestinal mucosal immune response. Intestinal microorganisms also play an important role in maintaining colonic homeostasis and local and systemic immunity (Rakoff-nahoum and Medzhitov, 2006; Li et al., 2008). Several studies have shown that filamentous bacilli closely adhered to intestinal epithelium can induce Th17 reaction and increase the number of Treg cells in the colon (Gaboriau-Routhiau et al., 2009). Simultaneously, single colonization of Bacillus fragilis can promote the proliferation of Treg cells and induce IL-10 production. Both of them can inhibit chemical-induced colitis (Round and Mazmanian, 2010). This suggests that microbial flora is driving the host immune response and reducing disease susceptibility. The correlation analysis of microbial flora and biochemical factors showed that the relative abundance of protective bacteria such as butyric acid bacteria, spirulina, lactic acid bacteria and bifid bacteria was positively correlated with anti-inflammatory cytokines such as IL-10, while the relative abundance of *Prevotella, rumen coccus, Bacteroides* and *Escherichia* were positively correlated with pro-inflammatory cytokines such as IL-23, TNF-α, IL-1β, IL-6, and IFN-γ (Pokusaeva et al., 2011).

In addition, short chain fatty acid (SCFA), such as acetate, propionate and butyrate, produced by microbial fermentation of polysaccharides, helps regulate intestinal homeostasis and may regulate gene expression through epigenetic regulation, thus reducing the production of pro-inflammatory factors in human adipose tissue. *Clostridium coccoides* is the main producer of SCFA, and can ferment polysaccharides to produce butyrate as the main energy source of colon cells. It has been proved that *Clostridium coccoides* can protect the colon from inflammatory damage (Donohoe et al., 2011; Brown et al., 2012). According to relevant studies, starch-embedded microspheres (containing polysaccharides) can regulate the content of SCFA and reduce the abundance of potentially harmful bacteria *in vitro* fermentation of fecal microorganisms in IBD patients, such as *Bacteroides vulgatus* and *Veillonella*. The specific mechanisms may include: starch-embedded microspheres show slow fermentation characteristics, which is conducive to increasing beneficial fermentation in the distal colon. The production of SCFA helps to maintain a relatively low colon pH, prevent potential harmful bacterial growth and reduce the activity of co-carcinogens such as glucuronidase, glycosidase and 7-α hydroxylase, produce butyrate as the main energy source of colon epithelial cells, and inhibit immune inflammation and downstream products by inhibiting the activation of transcription factor NF-κB as an anti-inflammatory agent. Such as pro-inflammatory cytokines IL-12 and TNF, and up-regulate the production of anti-inflammatory cytokine IL-10 (Rose et al., 2010). These studies have shown that polysaccharides can affect intestinal microorganisms and their metabolites, but the specific mechanism still needs to be further explored.

The fruit of *Schisandra chinensis* (Turcz.) Baill in the family of Magnoliaceae has been used as an herbal drug in traditional Chinese medicine for a long time (Liu, 1989). In particular, these fruits were widely used as health foods and medical products in the treatment and prevention of some chronic diseases, including gastrointestinal diseases, liver diseases and tumors (Choi et al., 2006; Panossian and Wikman, 2008). At present, a large number of studies have carried out qualitative and quantitative analysis of lignans and other components in *Schisandra chinensis* by mass spectrometry (Liu et al., 2017; Song et al., 2019; Su et al., 2020). However, the research on Polysaccharides in *Schisandra chinensis* is very limited and not deep enough. The polysaccharide is an important material base of *Schisandra chinensis* for anti-tumor and immune enhancement. The main goal of the present research is to investigate whether the anti-UC effect of SCP is related to the modulation of gut microbiota.

## Materials and Methods

### Extraction, Purification, and Physiochemical Analysis of SCP

Four hundred grams of the drying sample of *Schisandra chinensis* was added to the 3-fold amount of petroleum ether (1,200 mL) and then refluxed for 4 h. Then the petroleum ether was removed. After degreased, *Schisandra chinensis* was added to 8 times the weight of pure water (3,200 mL), which then was refluxed twice, 3 h for each time, and mixed together and filtered. The filtered extract was concentrated to 400 ml, after which the anhydrous ethanol was added until the extract reached 80% concentration and kept overnight. Finally, the crude polysaccharide of *Schisandra chinensis* was gained after centrifugation and filtration.

The crude polysaccharide of *Schisandra chinensis* was dissolved in 400 mL of pure water, then chloroform and n-butanol were added in proportion (water solution: chloroform: n-butanol = 25:5:1). The mixture was shaken sharply. Then it was allowed to stand and centrifuged, so that the impurities such as protein were removed. The remaining water phase was the polysaccharide solution. This process was repeated for five times. Finally, the refined polysaccharide of *Schisandra chinensis* (SCP, 21.6 g) was obtained by merging the water phases.

Five mg of refined polysaccharide was precisely weighed and dissolved in 1.0 mL trifluoroacetic acid (TFA, 2.0 mol/L). Then, 200 μL of the TFA solution was absorbed accurately and add the 10 ml plugged test tube. The tube was hydrolyzed in water bath at 100°C for 6 h and 200 μL methanol was added after cooling to room temperature. The process was repeated until the excess TFA is completely removed. Then 50 μL of NaOH solution (0.3 mol/L) was added to the tube to dissolve the residue completely, after which 50 μl of 1-phenyl-3-methyl-5-pyrazolone (PMP) solution (0.3 mol/L, dissolved in methanol) was added. The tube was placed at 70°C in a water bath for 100 min. After the reaction for 10 min, cooling to room temperature, 50 μl of hydrochloric acid (0.3 mol/L) was added in order to neutralize reaction, and water was added to replenish the solution to 1.0 mL. To discard chloroform layer, extraction of chloroform was repeated in equal volume for 3 times. Then after extracting 700 μl of water phase at 50°c, the solution was decompressed and concentrated. Finally, 600 μL of water was added to fully dissolve the residue, after which the solution was filtered by 0.45 μm microporous membrane and stored in refrigerator at 4°C.

### Mice and Ulcerative Colitis Model

A total of 24 male C57BL/6 mice (20 ± 2 g, aged ~8–10 weeks) were purchased from Shanghai Sipper-BK Lab Animal Co. Ltd. The mice were kept at constant conditions (24 ± 1°C and 60% humidity), which possess free tap water and rodent food at 12 h light/dark arrangement.

All the mice were allowed to acclimate for a period of 10 days before randomly divided into four groups with 8 mice each. Group I: The mice served as normal control (treated intragastric a ministration with normal saline for 3 weeks); Group II: The mice served as model received dextran sodium sulfate (DSS, MW 36,000–50,000; MP Biomedical) (10 mL/kg/body weight, daily); Group III: The mice received salazosulfapyridine (SASP) which is also the positive control group (200 mg/kg body weight, daily); Groups IV: The rat received SCP (8.0 g/kg body weight, daily, dissolved in pure water).

### Histological Examination of the Colon

The colon tissue about 0.5 cm long was cut, fixed with 10% neutral formalin, embedded in paraffin, sectioned, dewaxed and stained with hematoxylin and eosin (H & E). The changes of ulcer and inflammatory cell infiltration were observed under microscope. The histopathological score criteria of mice colon is as follows: 0: No obvious injury was found, 1: Damage to the epithelium of the colon, 2: Colonic mucosal ulcer, inflammatory cell infiltration of lamina propria, submucosal edema, 3: Transmural inflammation and ulcer, deformation, necrosis and exfoliation of colonic epithelium, 4: Transmural inflammation and ulcer, with normal mucosa, 5: Transmural inflammation and ulcer without normal mucosa.

### Cytokine Measurement of Colon

The activities of myeloperoxidase (MPO), reduced glutathione (GSH), nitrate (NO), superoxide dismutase (SOD), reactive oxygen species (ROS) and malondialdehyde (MDA) in colon were estimated spectrophotometrically using commercial ELISA kits.

In addition, the concentrations of TNF-α, IFN-γ, IL-1β, IL-4, IL-6, IL-10, IL-13, IL-17, and IL-23 inflammatory factors in colon tissues were assessed using commercial ELISA kits (Thermo Fisher, USA) in accordance with the manufacturer's instructions.

### Western Blot Analyses

The effect of SCP on the protein expression of IL-1β, IL-10, IL-23, TNF-α, and IFN-γ in the colon of DSS induced colitis mice was studied. The specific experimental methods are in the Supplementary Material.

### Microbial Diversity Analysis

We sequenced the 16S rRNA of 34 samples of intestinal contents of mice. The specific methods of DNA extraction, PCR amplification and sequencing data processing are in the Supplementary Material.

### Bioinformatics Analysis

Microbial differences analysis, alpha & beta-diversity analysis, correlation analysis, and co-occurrence network analysis were performed using I-sanger (Majorbio Bio-Pharm Technology Co. Ltd. Shanghai, China. www.i-sanger.com). The community diversity was evaluated by ace, sobs and shannon indices. A heat map based on the relative abundance of classification level of microbiota and genera were generated using R packages 2.15 in I-sanger. In addition, PCoA analysis based on species abundance was used to analyze the species composition and structure of intestinal flora in each group of mice.

### SCFAs Quantification

Standard solution preparation: acetic acid, propionic acid, butyric acid, isobutyric acid, valeric acid and isovaleric acid were precisely weighed and added into a 5 ml volumetric flask, and 0.005 M NaOH solution was added to the calibration. Standard mother liquor of 8.830, 5.820, 3.260, 1.586, 1.408, and 1.296 mg/mL was prepared, respectively.

Standard curve sample preparation: The mixed standard solution was obtained by eddy mixing of acetic acid, propionic acid, butyric acid, isobutyric acid, valeric acid and isovaleric acid with 300 μL, respectively. The mixed standard solution was diluted to the required concentration with 0.005 M NaOH solution. Furthermore, the mixed standard solution with 600 μL concentration was extracted and added into the EP tube containing 10 μL d3-caproic acid internal standard (630 μg/mL), respectively.

Pretreatment and Derivatization of Fecal Samples: The feces (100 mg) of each mouse was weighed and placed into a 2 mL centrifuge tube, and then 1 mL of 0.05 M NaOH aqueous solution was added, after which the mixture was homogenate for 10 min, centrifuge at 13, 200 rpm for 20 min at 4°C and whirled for 1 min and centrifuged (12,000 rpm/min, 20 min) at 4°C. Then 600 μL of the supernatant was absorbed and mixed in an EP tube containing 10 μL d3-caproic acid (630 μg/mL, internal standard). The above-mentioned fecal sample solution containing internal standard was added to a 10 mL glass centrifuge tube and 300 μL ultra-pure water was added to mix in a whirlpool. And then 500 μL 1-propanol/pyridine (3:2, v/v) and 100 μL propyl chloroformate were consecutively added. The mixture was swirled for 10 s and then ultrasonic for 1 min. After derivatization, the derivatives were extracted by adding 300 μL n-hexane. After 1 min of the scroll, centrifugation was carried out at 2,000 rpm for 10 min, and 200 μL of the upper solution was absorbed. Then 200 μL n-hexane was added to the glass centrifugal tube and the operation was repeated. Finally, the supernatant solution extracted twice was mixed evenly and centrifuged, and 200 μL of supernatant solution was absorbed for injection analysis.

In addition, all assays were performed using the Trace 1310-TSQ 8000 Evo GC-MS System (Thermo, USA). The separation of each compound was achieved using a Thermo TG-5MS capillary column (0.25 mm × 30 m, 0.25 μm). The initial oven temperature was 0°C, and then increased to 50°C by 25°C/min, 70°C by 10°C/min, 85°C by 3°C/min, 110°C by 5°C/min and finally 290°C by 30°C/min, which was then maintained for 8 min. The temperature of the ion source and injection port was set at 230°C and 290°C, respectively. The injected volume was 1 μL. The flow rate of helium was 1.2 mL/min with a 20:1 split ratio. For mass spectroscopy, electron bombardment ionization (EI) source was selected and the electron energy was 70 ev. A full scan and a sim scanning mode were adopted, the scanning range was 30–600 m/z. Carrier gas: high purity helium (purity > 99.999%).

### Statistical Analysis

Statistical analysis was performed using SPSS 19.0 (IBM, Armonk, New York), and all data were expressed as the mean ± standard deviation (SD). Comparisons between groups were analyzed using one-way ANOVA, and mapping with the GraphPad Prism® version 5.0 (GraphPad Software, San Diego, CA, USA). A level of probability of *p* < 0.05 was set as statistically significant.

## Results

### Yield and Physiochemical Characterization of SCP

SCP was obtained with a yield of 5.4% (w/w), a total carbohydrate content of which is 94.9%. SCP was composed of D-glucosamine, rhamnose, glucose, D-galactose, D-xylose and D-arabinose, with a molar ratio of 7.5: 1.4: 2.5: 79: 7.1: 2.5, respectively (Supplementary Material).

### Behavioral Expression

**Figure 1:**
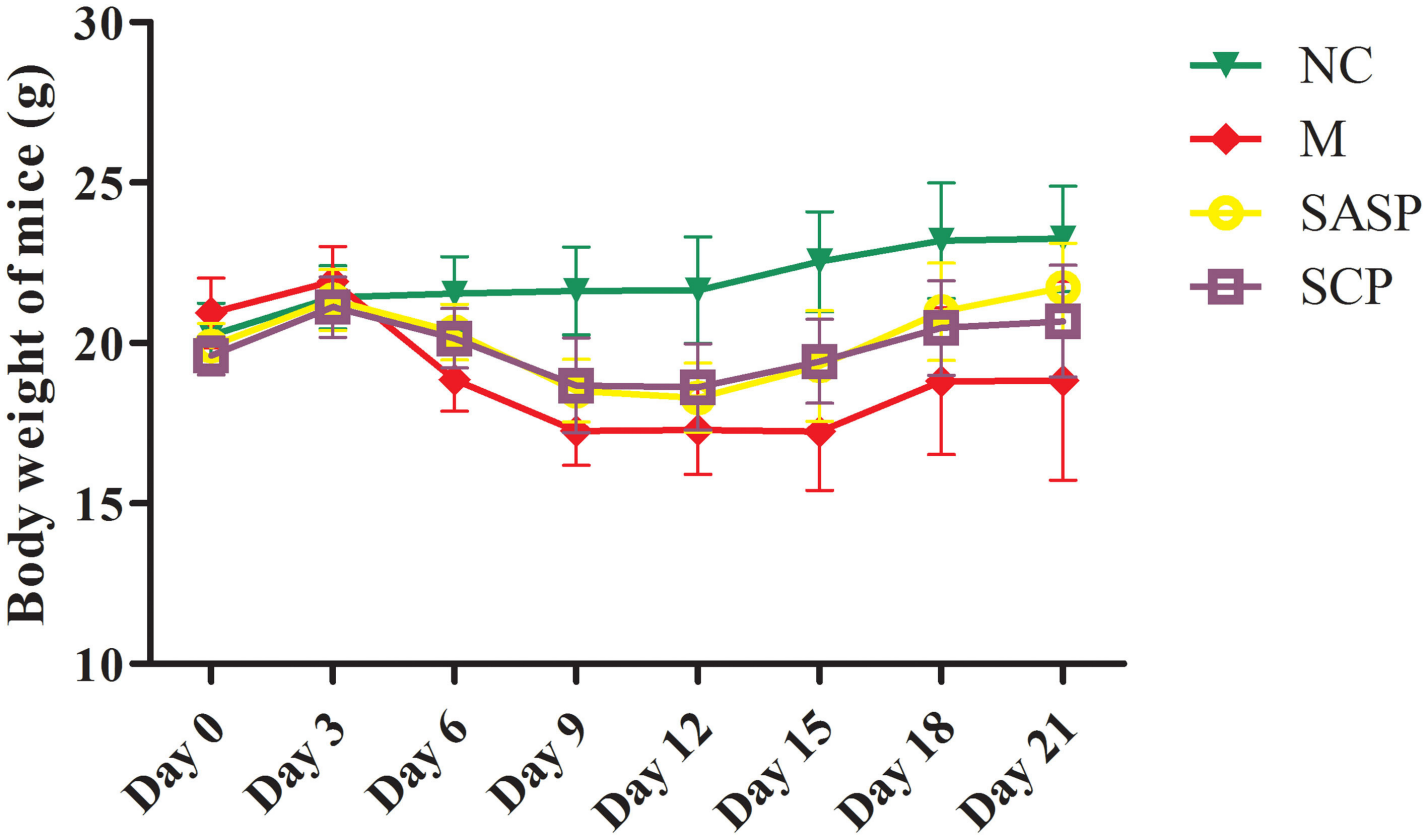
Effects of SCP on body weight. NC, control group; M, DSS group; SASP, positive drug group; SCP, *S. chinensis* polysaccharide group. The values are expressed as means ± S.D.

After the mice were induced by DSS, most of the mice showed physiological discomfort in varying degrees, such as mental malaise, diarrhea symptoms, and even bloody stool symptoms. The diarrhea, fecal occult blood and fecal blood symptoms were significantly improved when SASP and SCP polysaccharide were given. Weight loss is one of the most important indexes for the evaluation of DSS induced colitis. At the beginning of the experiment design, there were 8 mice in each group, but with the progress of the experiment, the DSS model mice would die, resulting in the difference in the number of mice between groups at the end of the laboratory. At the end of the experiment, the number of mice in each group was 8 in the NC group, 5 in the DSS group, 6 in the SASP group and 6 in the SCP group. The results (Figure 1) showed that the weight of model group (DSS induced colitis mice) was significantly lower than that of normal control group (*P* < 0.01). After SASP and SCP were given, the weight of colitis mice increased to a certain extent, especially SCP group (*P* < 0.05). In addition, in the process of modeling, researchers found that the weight of colitis mice began to decline from the third day. After giving drugs on the 7th day, the weight of colitis mice began to rise significantly from the 12th day (*P* < 0.05).

### Histopathological Analysis of Colon Tissues

**Figure 2:**
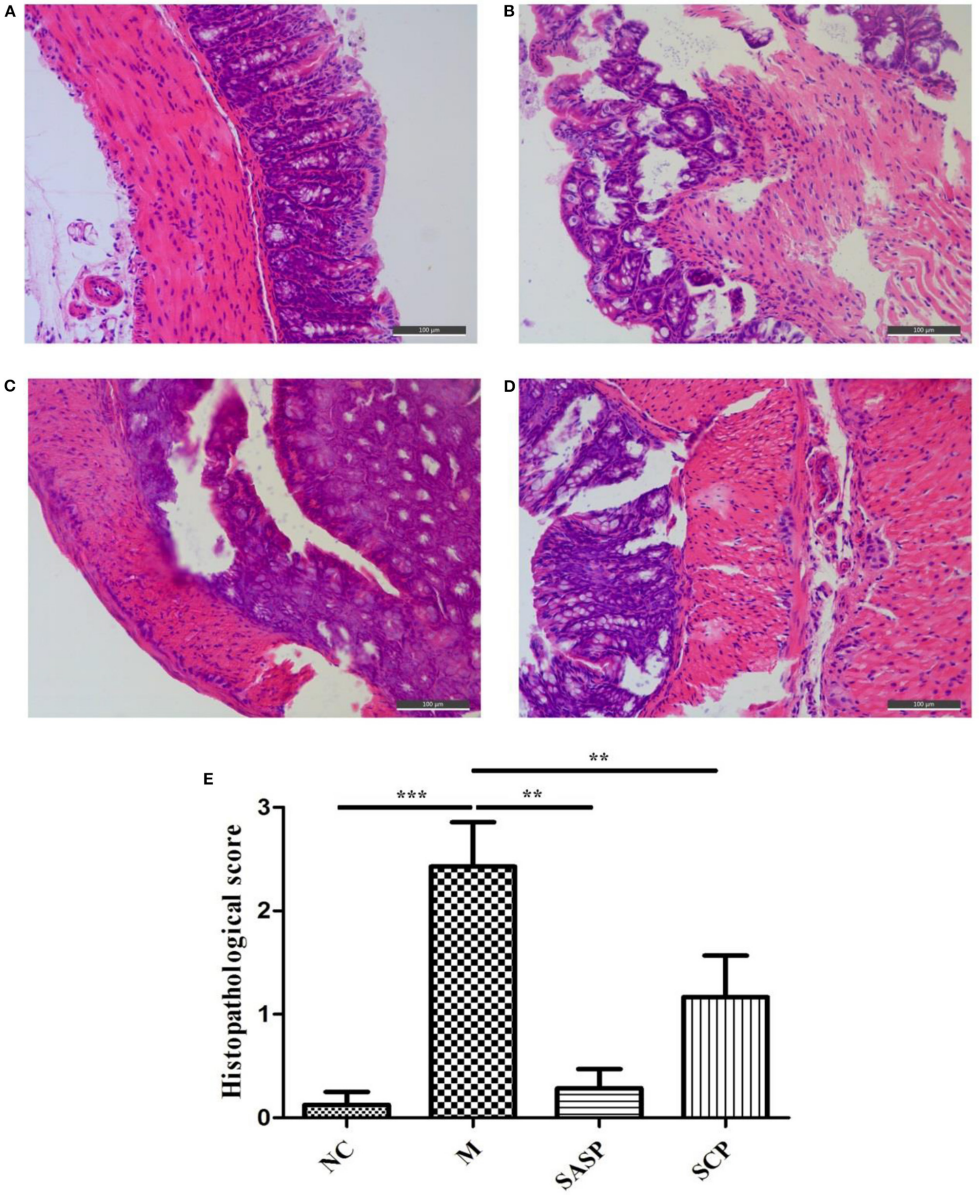
**(A–E)** Histopathological observation of the Colon. NC, control group; M, DSS group; SASP, positive drug group; SCP, *S. chinensis* polysaccharide group.

The histopathological changes in the colon in each group are shown in Figure 2. The results showed that in the NC group, the colonic mucosa epithelium was arranged in order, the crypt gland was obviously complete, the goblet cells were abundant, and there was no inflammatory cell infiltration; in the DSS model group, the colonic mucosa epithelium was ulcerated, the crypt structure was partially disappeared or deformed, the goblet cells were lost, and a large number of inflammatory cells were infiltrated. After SASP and SCP were given during the model period, the degree of colon damage in the model group was significantly lower than that in the model group, which indicated that SASP and SCP had a certain alleviation and protection effect on the occurrence of colitis, especially SCP.

### Changes of Inflammatory Cytokines

**Figure 3:**
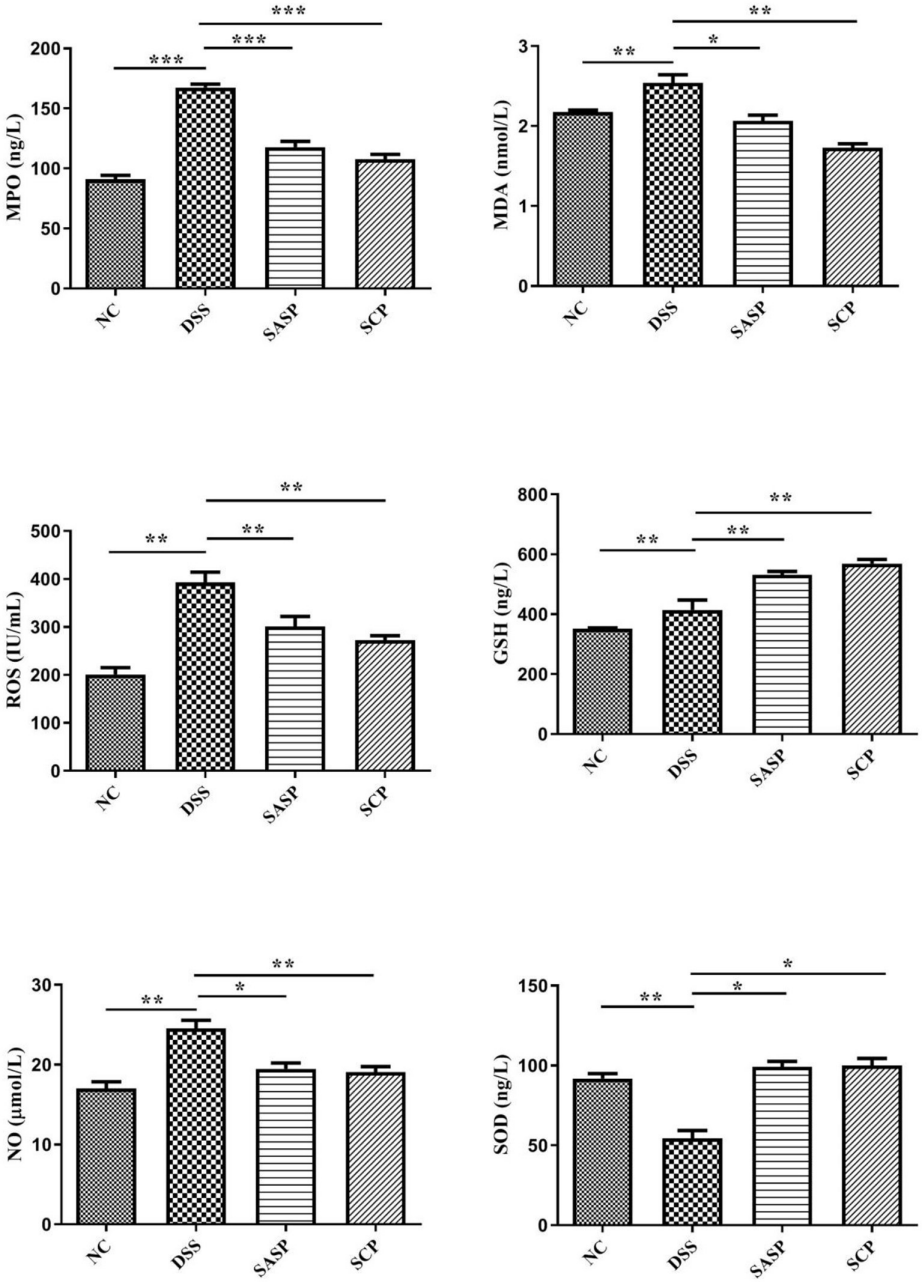
Levels of inflammatory cytokines. NC, control group; M, DSS group; SASP, positive drug group; SCP, *S. chinensis* polysaccharide group. Data are expressed as means ± S.D. *p < 0.05, **p < 0.01, ***p < 0.001.

**Figure 4:**
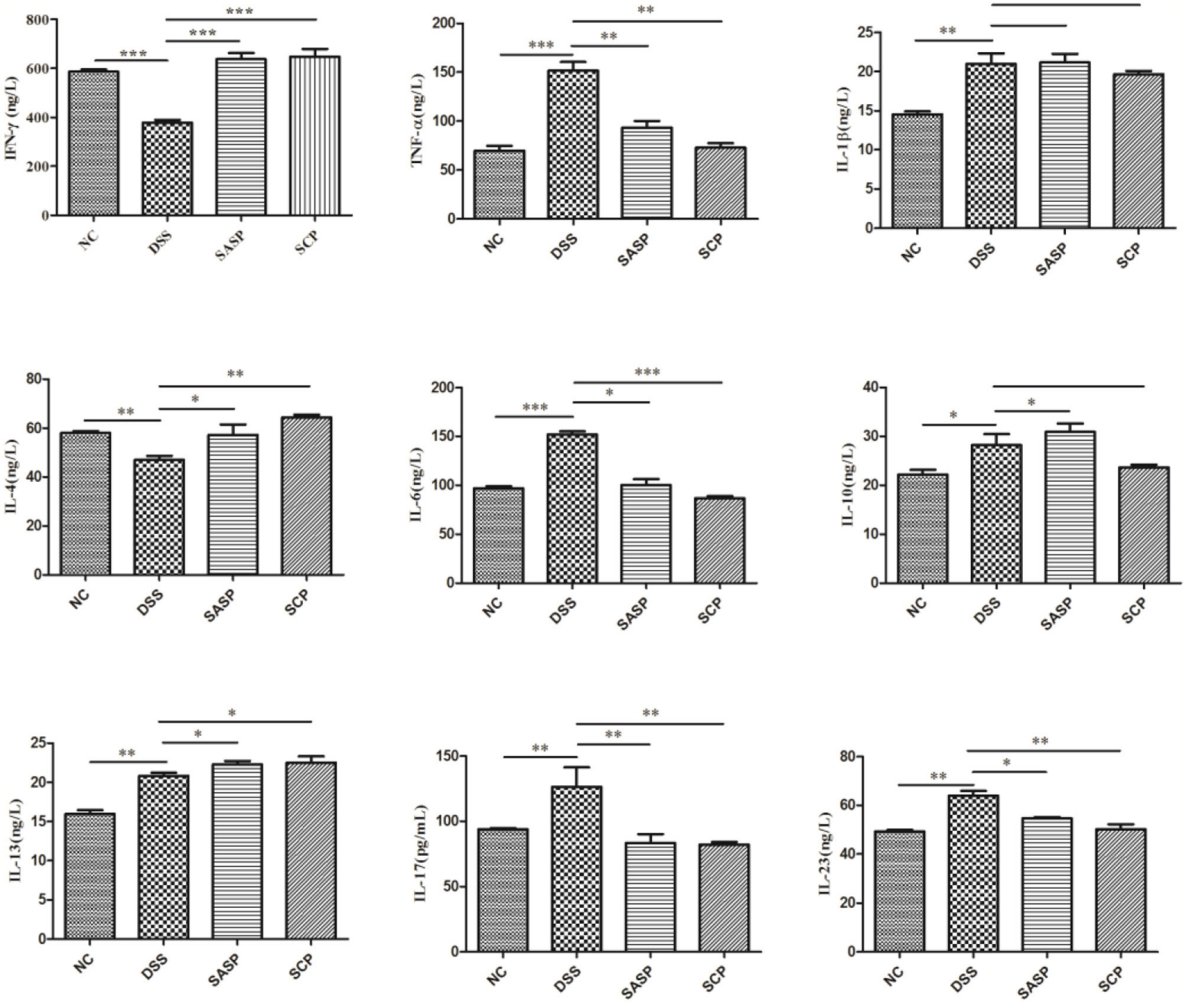
Levels of inflammatory cytokines. NC, control group; M, DSS group; SASP, positive drug group; SCP, *S. chinensis* polysaccharide group. Data are expressed as means ± S.D. *p < 0.05, **p < 0.01, ***p < 0.001.

To evaluate the effects of SCP on inflammation, the colon levels of MPO, GSH, NO, SOD, ROS and MDA, TNF-α, IFN-γ, IL-1β, IL-4, IL-6, IL-10, IL-13, IL-17, and IL-23 were measured. Compared to mice of the NC group, the levels of MPO, NO, ROS, MDA, TNF-α, IL-1β, IL-6, IL-10, IL-13, IL-17, and IL-23 in mice of the DSS group were significantly increased (*p* < 0.01), whereas the levels of GSH, SOD, IFN-γ, and IL-4 were decreased (*p* < 0.01) (Figures 3, 4). Compared to mice of the DSS group, SCP and SASP significantly decreased the levels of MPO, NO, ROS, MDA, TNF-α, IL-6, IL-13, IL-17, and IL-23, and increased the GSH, SOD, IFN-γ and IL-4 levels. However, there were no significant differences in the level of IL-1β and IL-10 (*p* > 0.05). Even SASP can increase the level of IL-10. Another quite valuable finding from the above results is that the SCP group is even more effective than the SASP group.

### The Proteins Expression of TNF-α, IFN-γ, IL-1β, IL-10, and IL-23

**Figure 5:**
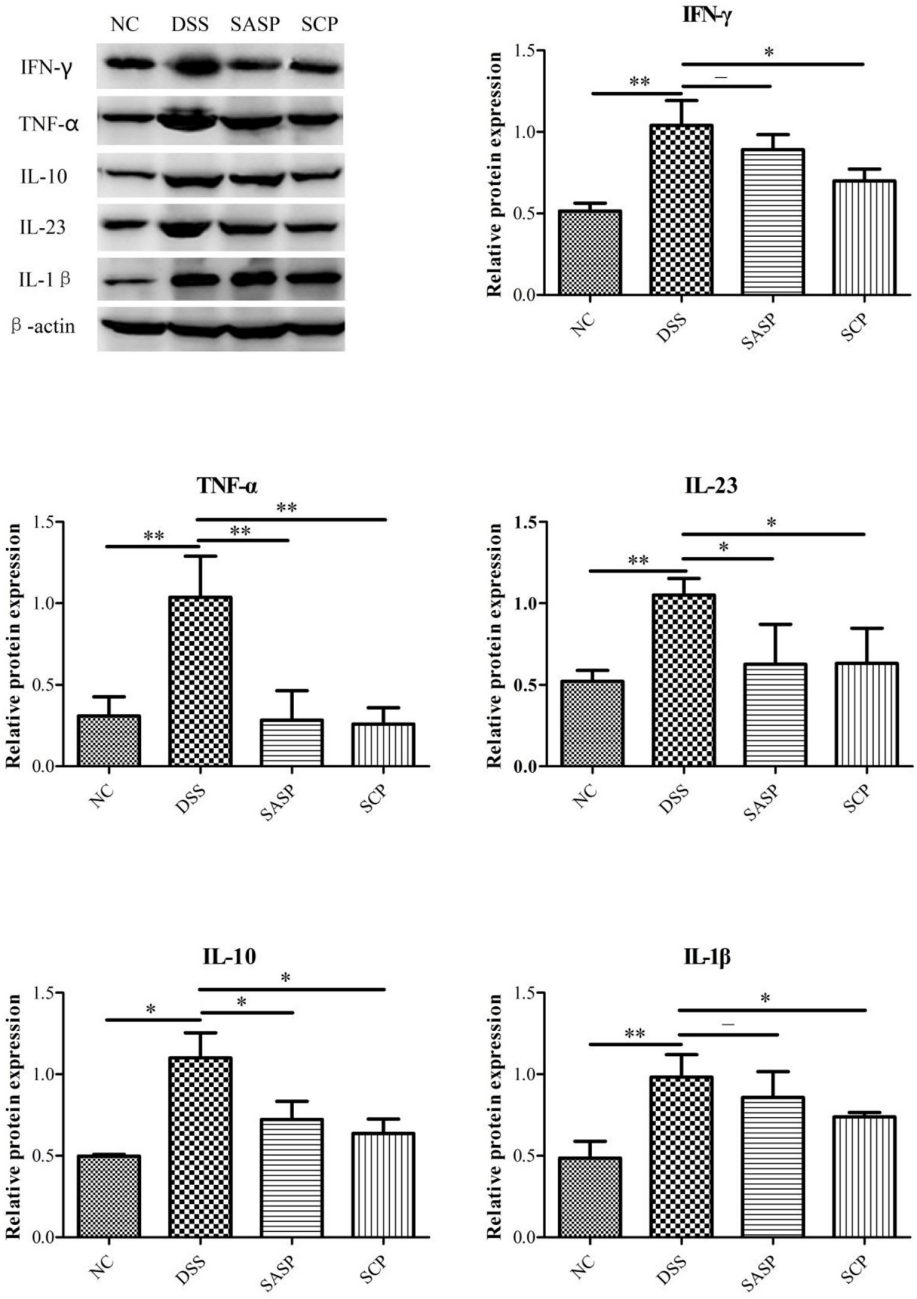
Expression level of key proteins. NC, control group; M, DSS group; SASP, positive drug group; SCP, *S. chinensis* polysaccharide group. Data are expressed as means ± S.D. *p < 0.05, **p < 0.01, ***p < 0.001.

We further examined the expression of inflammatory factors, including TNF-α, IFN-γ, IL-1β, IL-10, and IL-23, in the colon of each group by western blot analysis. As shown in Figure 5, the expressions of TNF-α, IFN-γ, IL-1β, IL-10, and IL-23 were markedly decreased in the mice of the DSS group, as compared with the NC group (*p* < 0.01; except for IL-10, *p* < 0.05). All treatment groups (SCP and SASP) could reduce the expression of the above proteins in varying degrees, that is, inhibit the inflammatory response of mice. Also, the SCP administration markedly inhibited the inflammatory response than SASP.

### Composition and Diversity of the Gut Microbiota

#### α and β Diversity Analysis of the Gut Microbiota

**Figure 6:**
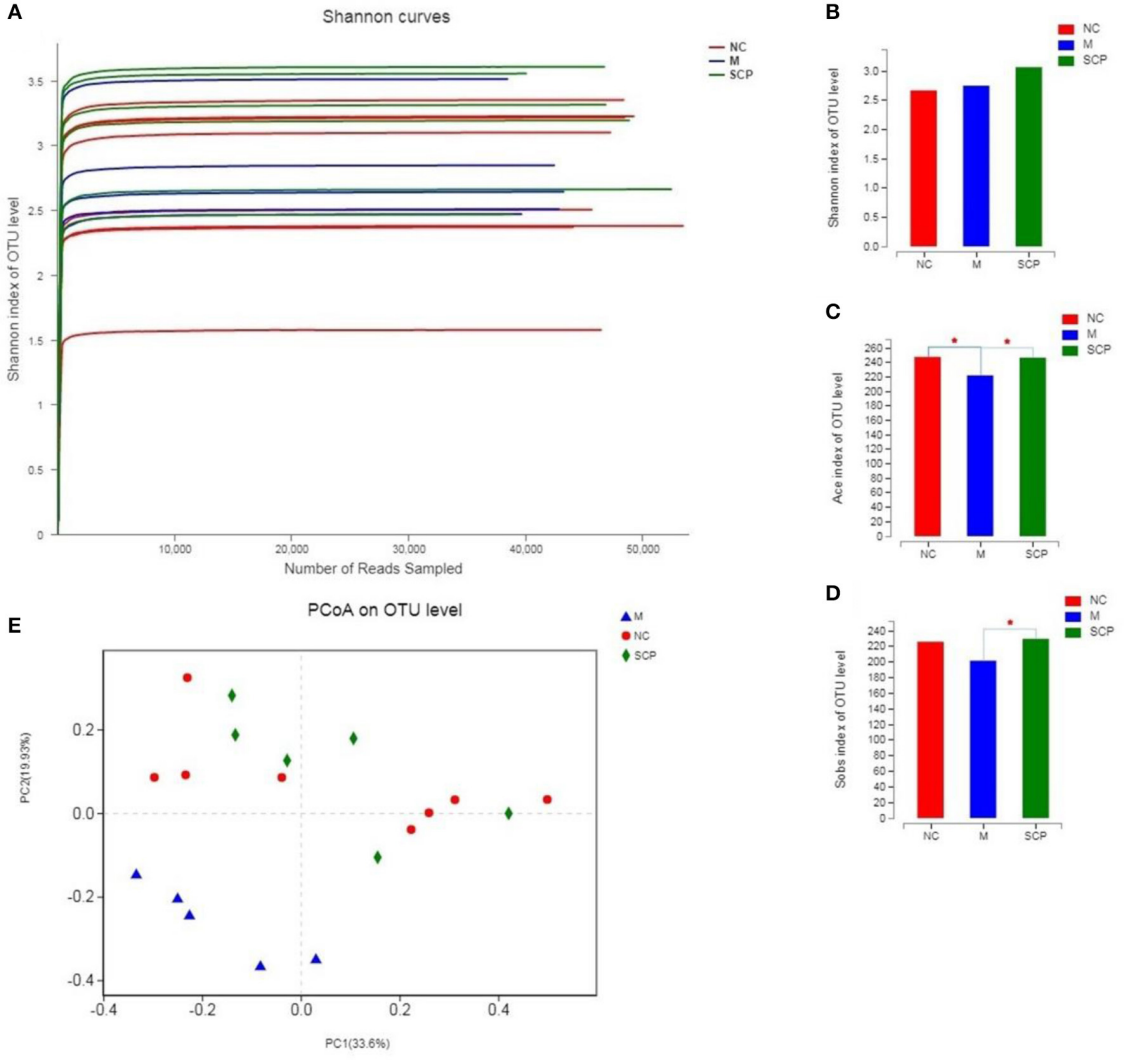
α and β Diversity analysis of the gut microbiota. **(A)** Rarefaction curves. **(B)** Sobs analysis. **(C)** Ace index. **(D)** Shannon index. **(E)** PCoA score plot. NC, control group; M, DSS group; SCP, *S. chinensis* polysaccharide group. Values are expressed as means ± S.D. Graph bars marked with different letters represent statistically significant results (*p* < 0.05) based on ANOVA with Duncan's range tests, whereas bars marked with identical letters represent no statistically significant differences.

The intestinal microbial diversity of mice was analyzed by the Illumina MiSeq System. The rarefaction curves and estimators are shown in Figure 6A. The rarefaction curves indicated that the sequencing depth of gut microbiota in each sample is fully captured and could be used for further analysis. The classification of species was calculated using the Sobs index, the community richness using the Ace and Shannon index (Figures 6B–D). The results showed that the community richness in SCP group significantly decreased as compared to DSS group (*P* < 0.05), however, the Shannon richness index of SCP was closer, albeit not significantly, to the NC group than the DSS group (*p* > 0.05). These indicate that increased inflammatory injury of colon in UC mice results in decreased microflora community richness, but has no influence on the community diversity. These results revealed that the richness and diversity of the gut microbiota in mice of the DSS group were significantly reduced compared with those of the NC group. These results also indicated that after the intervention of SCP, the species number, abundance and coverage of intestinal flora were improved. SCP has a significant regulatory effect on the intestinal flora of colitis mice, which may be one of the important mechanisms of its anti-ulcerative colitis.

Principal coordinate analysis (PCoA) was used to evaluate the clustering of the gut microbiota in each group (Figure 6E). A plot of the PCoA scores showed that the DSS group had a significant shift along the PC1 compared with NC and SCP groups. SCP group showed a slight structural shift along the PC2. The location of the SCP group was closer to NC as compared to the DSS group. PCoA indicated a significant difference in the bacterial community between SCP group and DSS group.

#### Composition Analysis of the Gut Microbiota

**Figure 7:**
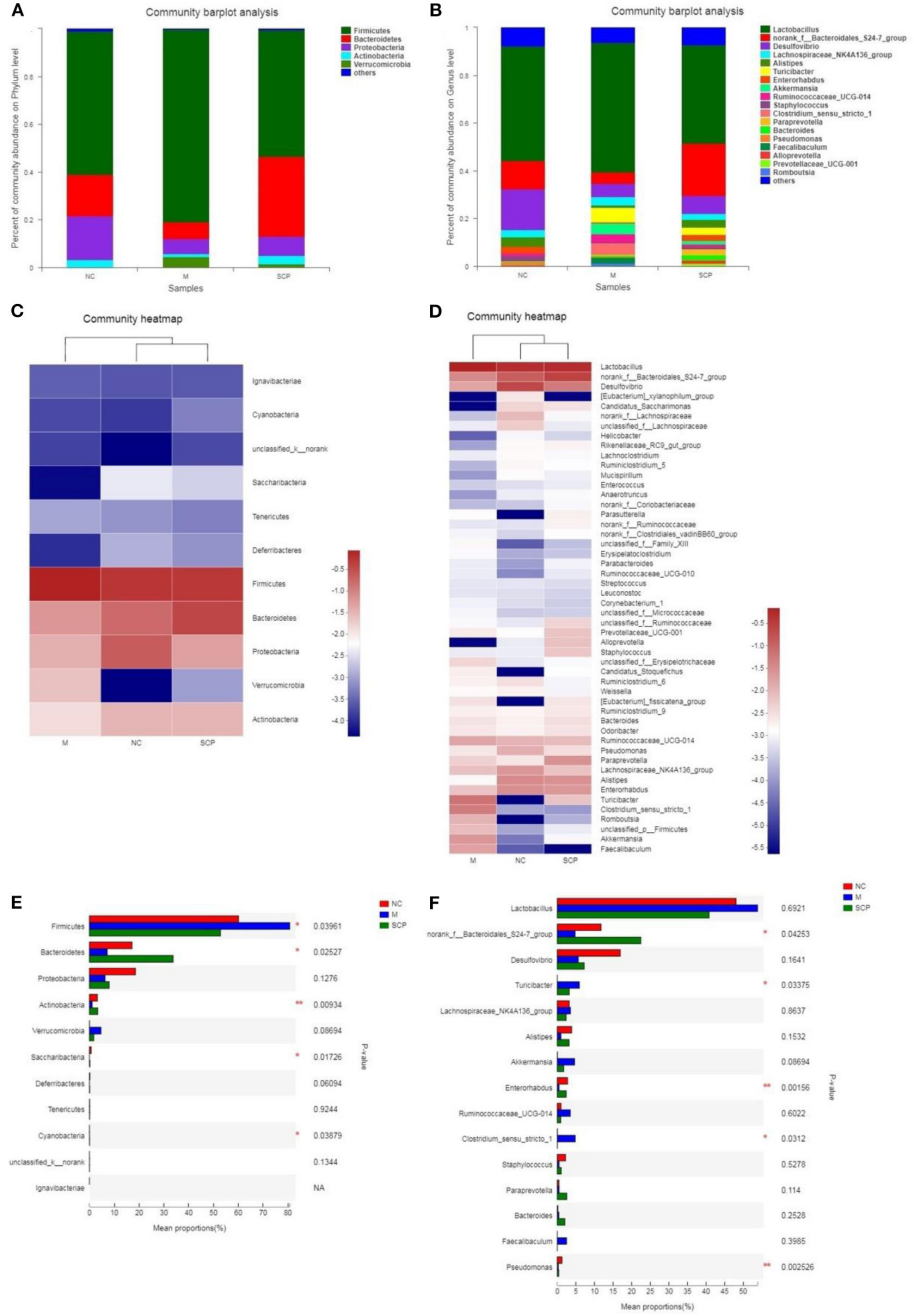
Composition of the gut microbiota. **(A)** Phylum level. **(B)** Genus level. **(C)** Heat map analysis at phylum level. **(D)** Heat map analysis at genus level. **(E)** Main different composition at phylum level. **(F)** Main different composition at genus level. NC, control group; M, DSS group; SCP, *S. chinensis* polysaccharide group.

The microbiota composition in different groups was further investigated. The relative abundance of the gut microbiota classification units was presented as a stacking histogram (Figure 7). At the phylum level, all the groups mainly composed of *Firmicutes, Bacteroidetes, Proteobacteria*, and *Actinobacteria* (Figure 7A). Although DSS treatment induced a significant decrease in the relative abundance of *Bacteroidetes, Proteobacteria*, and *Actinobacteria*, there was a dramatic increase in *Firmicutes*. SCP improved gut dysbiosis. After SCP treatment, the relative abundance of *Firmicutes, Proteobacteria*, and *Bacteroidetes* returned to their respective normal levels. There was no significant difference in the main components between SCP and DSS groups at the phylum level. At the genus level (Figure 7B), DSS treatment induced a significant decrease in the relative abundance of *norank_f_Bacteroidales_S24-7_group, Desulfovibrio* and *Alistipes*, there was a dramatic increase in *Lactobacillus, Turicibacter* and *Clostridium_sensu_stricto-1*. The proportion of *norank_f_Bacteroidales_S24-7_group, Desulfovibrio* and *Alistipes* significantly increased and significantly decreased *Lactobacillus, Turicibacter* abundance after SCP treatment. In addition, at the taxonomic level of phylum and genus, we also counted the species abundance of each sample, and studied the community composition intuitively through the visualization method of the Heatmap (Figures 7C,D). The results showed that SCP and NC groups could be clustered well at phylum and genus levels, and could be significantly separated from DSS groups. This revealed that the intestinal flora composition of mice treated with SCP was closer to that of NC group.

The changes in the relative abundance of each phylum and genus among groups were further compared (Figures 7E,F). At phylum levels, the genera with a higher ratio of abundance were *Firmicutes* and *Verrucomicrobia* in the DSS group. Compared to the DSS group, SCP increased the relative abundance of *Bacteroidetes, Actinobacteria*, and reduced the relative abundance of *Firmicutes* and *Verrucomicrobia*. At genus levels, compared to the NC group, the abundance of *norank_f_Bacteroidales_S24-7_group, Desulfovibrio, Alistipes, Enterorhabdus* and Pseudomonas in DSS group were decreased significantly, and the abundance of *Tericibacter, Akkermansia, Ruminococcaceae_UCG-14* and *Clostridium_sensu_stricto_1* in DSS group were increased significantly. Compared to the DSS group, SCP can regulate the above gut microbiota to the level close to the NC group.

**Figure 8:**
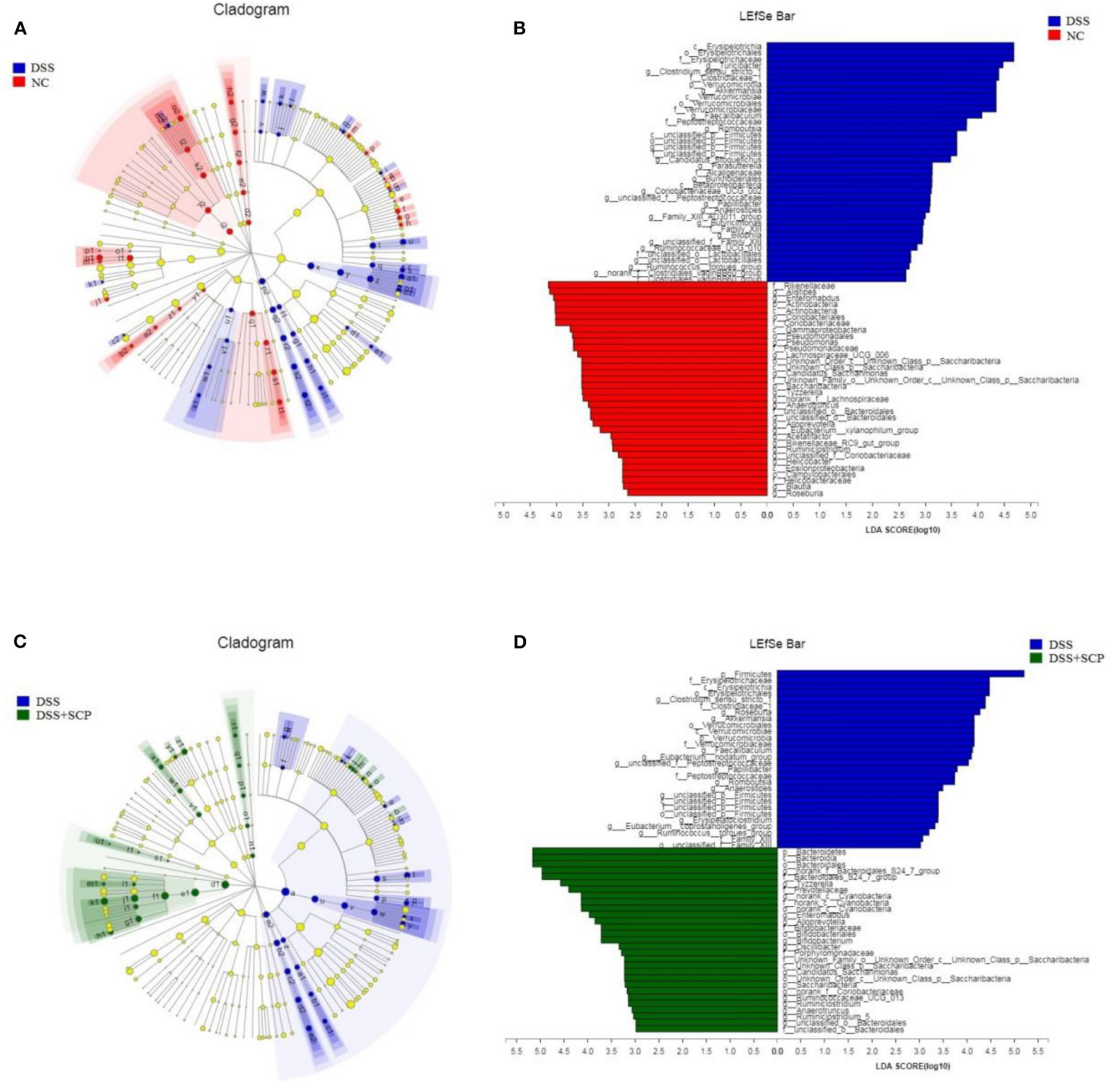
**(A–D)** Cladograms indicating the polygenetic distribution of bacterial lineages associated with the different groups. Indicators between inner and outer mangrove sediments with LDA score larger than 2.5. LEfSe provided the features that are differential bacterial taxa ranking according to the effect size. Different color nodes represent the microbial groups that are significantly enriched in corresponding groups and have significant influence on the differences between groups. The light yellow node indicates the microbial groups that have no significant difference in different groups or have no significant influence on the differences between groups.

Linear discriminate analysis (LDA) effect size (LEfSe) is a statistical tool designed to find biomarkers from metagenome data with default parameters to identify potential discriminating taxa between two groups. An LDA score of 2.5 was used for identifying bacterial groups with statistical significance. When the DSS group was compared with the NC group, 38 taxa with significant differences were found in DSS group, and 35 taxa showed significant abundance in NC group (Figures 8A,B). The clade graph showed that the difference contribution degree of *Verrucomicrobia* in DSS group was higher at the phylum level, and the difference contribution degree of *Actinobacteria* and *Saccharibacteria* in NC group was higher at the phylum level. The species and abundance of different species in DSS group were significantly higher than those in NC group. When the SCP group was compared with the DSS group, 28 taxa with significant differences were found in DSS group, and 29 taxa showed significant abundance in SCP group (Figures 8C,D). The results showed that the difference contribution degree of *Fimicutes* and *Verrucomicrobia* in DSS group was higher at the phylum level, and the difference contribution degree of *Bacteroidetes* and *Saccharibacteria* in SCP group was higher at the phylum level. Furthermore, the difference contribution degree of *Clostridium_sensu_stricto_1, Roseburia, Akkermansia, Faecalibaculum, Eubacterium_nodatum_group* etc. in DSS group was higher at the genus level, and the difference contribution degree of *norank_f_Bacteroidales_S24-7_group, Tyzzerella, norank_c_Cyanobacteria, Enteromabdus, Alloprevotella* etc. in SCP group was higher at the genus level.

Results from LEfSe showed that there were 73 taxa that could distinguish the DSS group from the NC group, and 57 taxa that could distinguish SCP group from DSS group with the LDA score > 2.5. In SCP group, the abundances of *Alloprevotella* genus from *Bacteroidetes* phylum (*P* = 0.005062), *Saccharibacteria* phylum (*P* = 0.005712), *Bacteroidetes* phylum (*P* = 0.004075), and *Bacteroidales_S24_7_group* family (*P* = 0.01762) from *Bacteroidetes* phylum increased significantly as compared to DSS group. But the abundances of *Anaerotruncus* genus (*P* = 0.006170) from *Firmicutes* phylum and *Firmicutes* (*P* = 0.02846) phylum were higher in DSS group.

### Analysis of Fecal SCFAs

**Figure 9:**
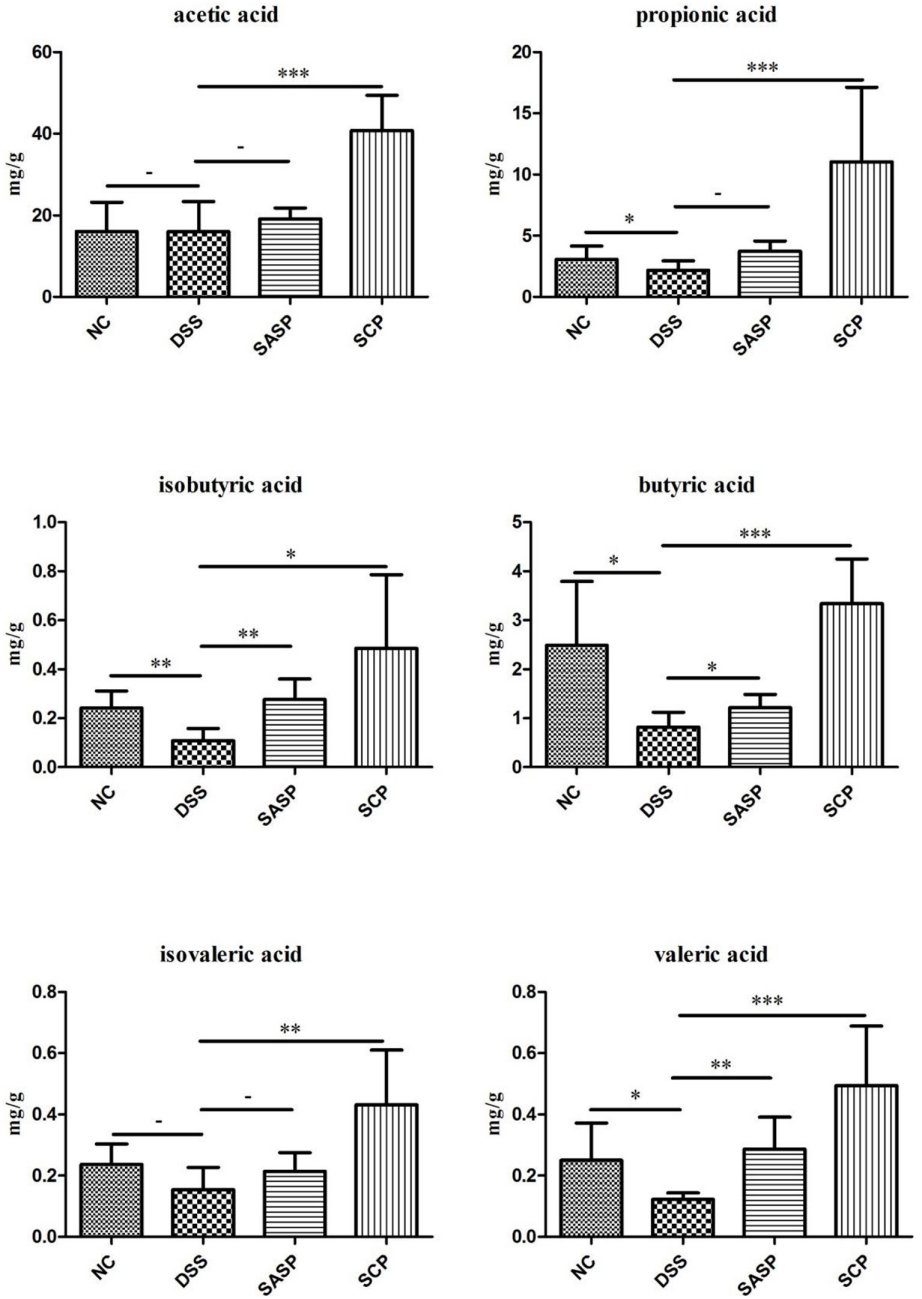
Concentrations of SCFAs in mice colonic contents. NC, control group; M, DSS group; SASP, positive drug group; SCP, *S. chinensis* polysaccharide group. Data are expressed as means ± S.D. * 0.01 < *p* ≤ 0.05, ** 0.001 < *p* ≤ 0.01, *** *p* ≤ 0.001.

The content of acetic acid, propionic acid, butyric acid, isobutyric acid, valeric acid and isovaleric acid was investigated to evaluate the effects of SCP on microbial metabolites (Figure 9). After the mice were treated with DSS, the content of propionic acid, butyric acid, isobutyric acid, and valeric acid significantly decreased in the colonic contents in mice of the DSS group compared with those of the NC group (*p* < 0.05). The SCFAs levels in mice of the SCP group recovered at different levels, especially the propionic acid, butyric acid and valeric acid levels (*p* < 0.001). However, the recovery of the SCFA level after SASP administration was not significant, except isobutyric acid and valeric acid.

### Correlation Between Gut Microbiota and SCFAs

**Figure 10:**
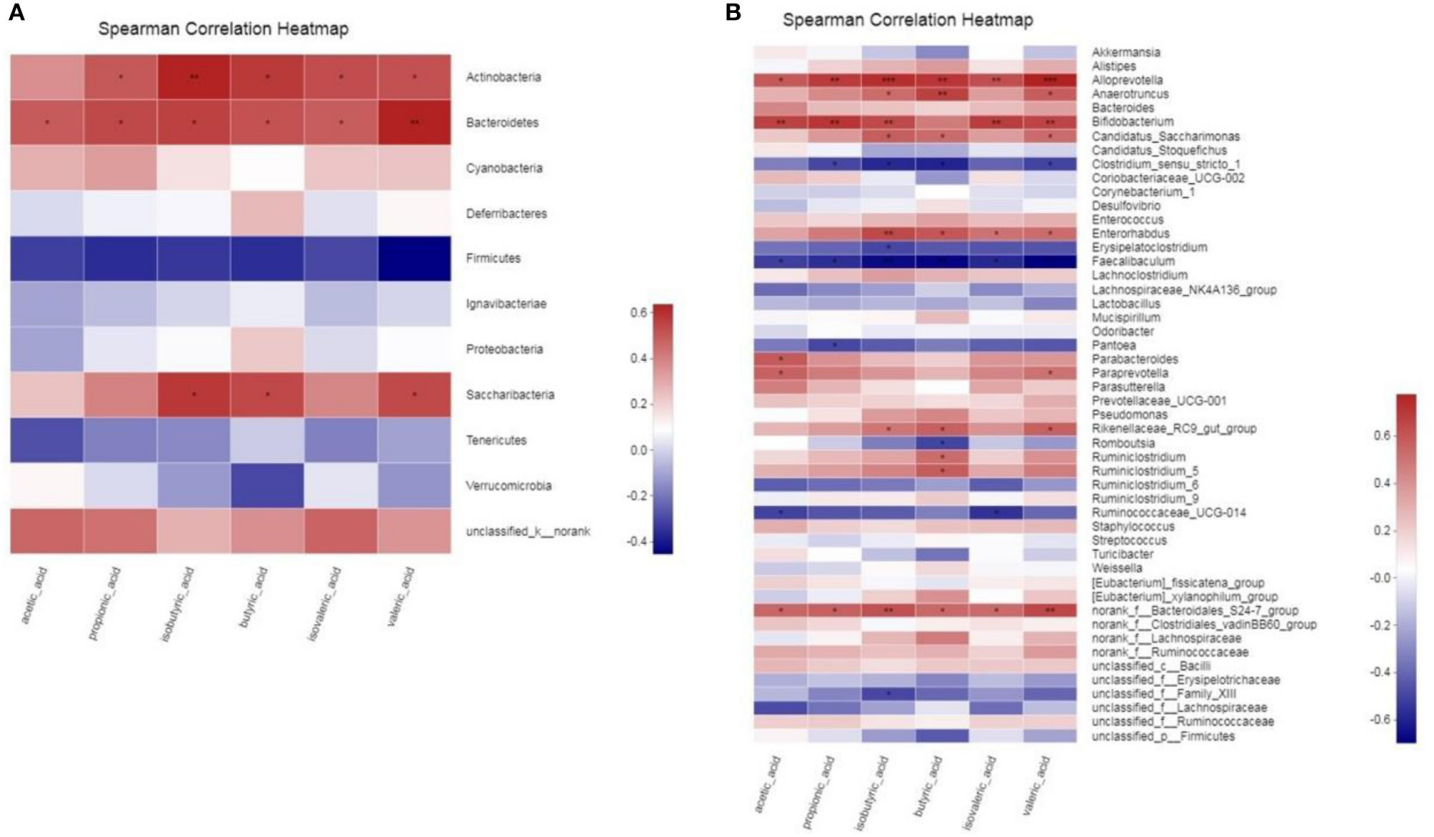
**(A,B)** Correlation between gut microbiota and SCFAs parameters. * 0.01 < *p* ≤ 0.05, ** 0.001 < *p* ≤ 0.01, *** *p* ≤ 0.001.

To determine whether there is a potential association between the alteration of the gut microbiota and host metabolism, we analyzed the correlation between the relative abundance of the gut microbiota and the SCFAs using Spearman correlation analysis (Figure 10). At the Phylum level, acetic acid, propionic acid, butyric acid, isobutyric acid and isovaleric acid all had a significant positive correlation with *Bacteroidetes* (*p* < 0.05). There was a significant positive correlation between valeric acid and *Bacteroidetes* (*p* < 0.01). And propionic acid, butyric acid, valeric acid and isovaleric acid all had significant positive correlation with *Actinobacteria* (*p* < 0.05). There was a significant positive correlation between isobutyric acid and *Actinobacteria* (*p* < 0.01). In addition, butyric acid, isobutyric acid and valeric acid all had significant positive correlation with *Saccharibacteria* (*p* < 0.05). At Genus level, SCFAs main had significant positive correlation with *Alloprevotella, Anaerotruncus, Bifidobacterium, Candidatus_Saccharimonas, Enterorhabdus, Rikenellaceae_RC9_gut_group* and *norank_f_Bacteroidales_S24-7_group* (*p* < 0.05), and main had significant inverse correlation with *Clostridium_sensu_stricto_1, Faecalibaculum* and *Ruminococcaceae_UCG-014* (*p* < 0.05). The statistical results showed that butyric acid, isobutyric acid and valeric acid had the highest correlation with gut microbiota.

## Discussion

Ulcerative colitis is a chronic recurrent disease characterized by intestinal inflammation and epithelial injury (Van Der Veen et al., 2009). In this study, we successfully established a UC mouse model. As a result, the intestinal microbial structure was destroyed, the level of inflammatory cytokines increased, the intestinal microbial environment was unbalanced, and the concentration of SCFAs in the intestinal contents decreased. More and more evidence shows that the complex interaction between TCM and the intestinal micro-ecosystem is essential during the treatment of TCM (Xu et al., 2017). In this study, *Schisandra chinensis* polysaccharide improved the composition and diversity of the gut microbiota by treating UC mice.

The changes of cytokines are also related to the treatment of SCP, such as interleukin and tumor necrosis factor play an important role in the immune system. As communicators between immune cells, they can reflect the host's inflammation. IL-1β, IL-4, and TNF-α levels were negatively correlated with cereal fiber intake (Chuang et al., 2011). Tn cells differentiate into Th1 cells induced by IL-12. Th1 cells specifically express T-bet and secrete IFN - γ. IFN - γ is associated with the pathogenesis of organ-specific autoimmune diseases (Hirahara and Nakayama, 2016). Tn cells are differentiated into Th2 cells by the action of IL-4 to protect the body from pathogens. Th2 cells can also be regulated by the transcription factor GATA-3 and secrete IL-4, IL-5, IL-13 and other effector cytokines to regulate allergic reaction (Choy et al., 2015). Cytokines such as IL-6, IL-23, TGF - β and IL-1 β induce TN cells to differentiate into Th17 cells, mediate the inflammatory response, eliminate bacteria and fungi outside the cells, and are closely related to the occurrence and development of autoimmune diseases. Tregs cells were induced by TGF - β alone to promote tissue repair. Tregs cells express forked head transcription factor Foxp3 and secrete IL-10, which can inhibit the inflammatory response (Joller et al., 2014). In this study, mice of the DSS group showed increased levels of IL-1β, IL-6, IL-10, IL-13, IL-17, IL-23, and TNF-α, but decreased levels of IFN - γ and IL-4, suggesting that UC associated with these changes in inflammatory cytokines. Compared to the DSS group, SCP significantly increased the IFN - γ and IL-4 levels; but decreased IL-6, IL-10, IL-17, IL-23, and TNF-α levels significantly. SCP might possess anti-inflammatory ability, which explains why the recovery in the cytokine levels was closer to the NC group. Intestinal disorders are manifestations of a variety of diseases, especially metabolic syndromes such as obesity, diabetes, hypertension, and hyperlipidemia (Thomas et al., 2017). It has been reported that *Schisandra chinensis* polysaccharides can improve antibiotic-related diarrhea in rats by reducing the relative abundance of *Blautia, Enterobacteriaceae*, and *Lachnospiraceae-UCG-008*. But at the genus level, the relative abundance of *Rumenococcus-1, Rumenococcus-UCG-014* and *Erysipelatoclostridium* were reduced (Qi et al., 2019). In this study, the gut microbiota richness and diversity of the NC group and the DSS group were significantly different. The Sobs, Ace and Shannon richness index results showed that the recovered gut microbiota richness of SCP was closer to NC mice. The composition analysis of the gut microbiota indicated that mice of SCP group basically eliminated the dysbacteriosis caused by DSS.

SCFAs are important metabolites in the gut microbial environment. They are also closely involved in immune, anti-tumor and anti-inflammatory activities (Cousens et al., 1979; Frost et al., 2014; Fernández et al., 2016; Li et al., 2018). A change in metabolite levels can reflect the homeostasis of the gut microbiota. The latest research shows that SCFAs can not only effectively reduce the incidence of enteritis, cardiovascular disease, colon cancer, obesity and diabetes, but also play an important role in maintaining the balance of energy metabolism (mainly glucose metabolism) and increasing insulin tolerance (Delzenne and Cani, 2005; Delzenne et al., 2011). It has been reported that propionate and butyrate can activate intestinal gluconeogenesis through the “gut-brain axis” neural network, maintain body weight balance and regulate normal blood glucose level (De Vadder et al., 2014). During UC, the production of SCFAs suddenly decreased. We found that SCP significantly improved the content of propionic acid, butyric acid and valeric acid in the colon of treated mice, especially the butyric acid. Butyric acid plays an important role in maintaining the stability of intestinal microecology. It can provide energy for host intestinal epithelial cells, especially in colon and cecum (Vinolo Marco et al., 2011). On the other hand, butyric acid can improve the structure of bacterial population, improve intestinal immunity and maintain intestinal homeostasis (Furusawa et al., 2013; Dong et al., 2016). Some studies have shown that butyrate can activate peroxisome proliferator activated receptor - γ (PPAR - γ) in colon cells, inhibit the expression of NOS2, reduce the level of nitrate, and consume oxygen by promoting β - oxidation of colon cells, thus avoiding the growth and proliferation of pathogenic bacteria (Byndloss et al., 2017). Gut microbiota and its metabolites are very important for the host's immune regulation. As an important immune regulatory molecule, they can regulate the production, transfer and function of immune cells (Goncalves et al., 2018). It was found that butyric acid can also activate GPR to affect the activation of inflammatory factors such as IL-1 and IL-6, inhibit the expression of other inflammatory factors, and promote the secretion of intestinal antimicrobial peptides and the apoptosis of T cells (Aguilar et al., 2014; Ran et al., 2018). Two biological activities of butyric acid in the colon (inhibition of proliferation of colonic epithelial stem cells and inflammation) are related to the inhibition of histone deacetylase activity (Verma et al., 2018). In addition, butyric acid, as a histone deacetylase inhibitor, enhances the intestinal mucosal immune response in the proliferation and differentiation of T and B lymphocytes (Park et al., 2015; Kim et al., 2016), which may be related to butyric acid driving the differentiation and function of macrophages, increasing the expression of antimicrobial peptides and enhancing the bacteriostatic ability after bacteriostatic ability after inhibiting histone deacetylase-3 (Schulthess et al., 2019).

In present study, SCP is prepared by boiling in water, and it may not be the most effective method for extracting polysaccharide from *Schisandra chinensis*. The emerging potential methods of pretreatment and extraction of active components with ammonia and hydrogen peroxide (Zhao et al., 2017a, 2020a,b) and pre-soaking (Zhao et al., 2017b; Qiao et al., 2018) may bring unexpected insights into the material basis of *Schisandra chinensis*. In addition, *Schisandra chinensis* contains water, and the determination of water content may be performed before extraction (Zhang et al., 2019), which contributes to accurate determination of polysaccharide in future research.

In conclusion, we found SCP had beneficial effects on mice with UC by recovering the gut structure, adjusting the cytokine levels, improving the diversity and composition of the gut microbiota and increasing the production of SCFAs. Compared to mice of the DSS group, SCP ameliorated the colon levels of IFN-γ, TNF-α, IL-1β, IL-10, IL-23, IL-4, IL-17, IL-13, and IL-6, adjusted the relative abundance of *norank_f_Bacteroidales_S24-7_group, Desulfovibrio, Alistipes, Lactobacillus, Turicibacter, Firmicutes, Proteobacteria* and *Bacteroidetes* at the genus and phylum level and significantly increased the content of acetic acid, propionic acid, butyric acid and total SCFAs. Our results indicated that SCP might serve as a potential natural product to treat UC.

## Data Availability Statement

The datasets generated for this study can be found in NCBI Bioproject with accession number: PRJNA658862, https://www.ncbi.nlm.nih.gov/bioproject/PRJNA658862.

## Ethics Statement

The animal study was reviewed and approved by Experimental Animal Center of Nanjing University of Chinese Medicine (license No. AEWC-20190705-80).

## Author Contributions

LS: conceptualization, methodology, software, and roles/writing—original draft. CM: data curation, funding acquisition, and formal analysis. XW: visualization, investigation, and methodology. LL: supervision and validation. HT: software and resources. JM: data curation. DJ: investigation, writing—review, and editing. TL: project administration, writing—review, and editing. MH and ZH: data curation and formal analysis. CF: visualization, writing—review, and editing. KZ and GY: software. All authors contributed to the article and approved the submitted version.

## Conflict of Interest

The authors declare that the research was conducted in the absence of any commercial or financial relationships that could be construed as a potential conflict of interest.
