# Supplementary material for: The Anti-colitis Effect of Schisandra chinensis Polysaccharide Is Associated With the Regulation of the Composition and Metabolism of Gut Microbiota
Source: Front Cell Infect Microbiol. 2020 Oct 21;10:519479. doi: 10.3389/fcimb.2020.519479 (PMC7609416; doi:10.3389/fcimb.2020.519479)
Supplement: Supplementary file 2 [file Table_2.DOCX]

**1. Monosaccharide composition and infrared spectrum analysis of *Schisandra chinensis* polysaccharide**

**1.1 Monosaccharide composition of SCP**


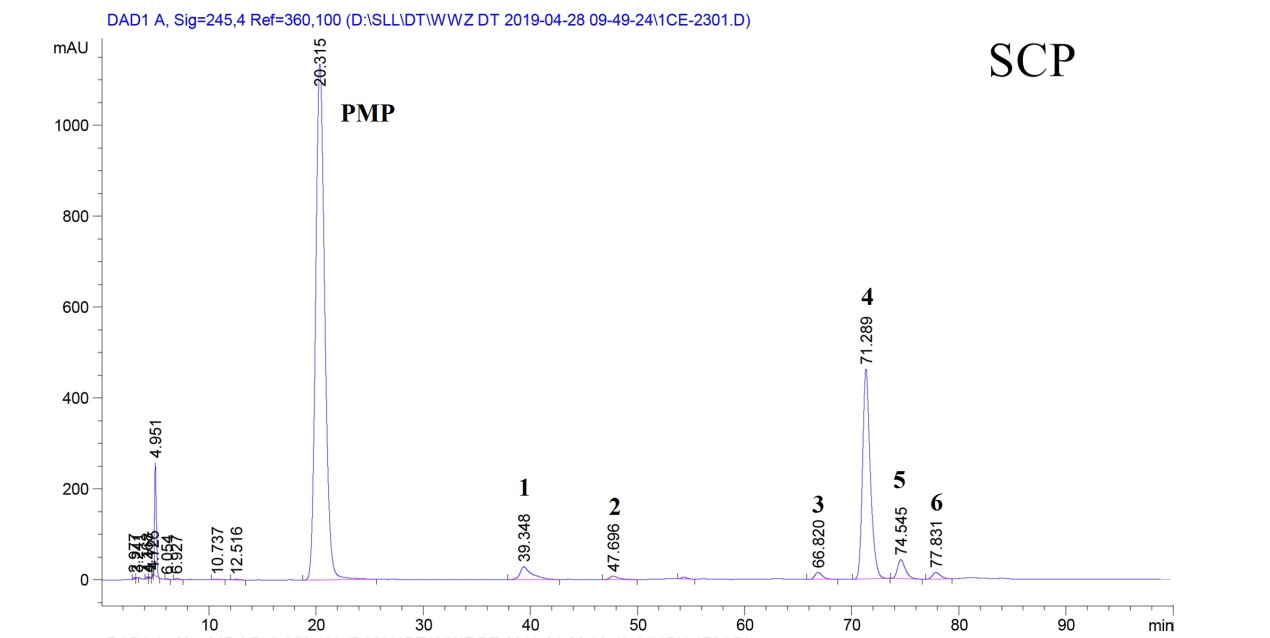


**Figure 1.** Monosaccharide composition of SCP. Peak 1: D-glucosamine, Peak 2: rhamnose, Peak 3: glucose, Peak 4: D-galactose, Peak 5: D-xylose, Peak 6: D-arabinose.

**1.2 Infrared spectrum analysis of SCP**

According to the infrared spectrum of SCP in 400 to 4000 cm^-1^, as shown in Figure 2. The results showed that 3408 cm^-1^ was the stretching vibration peak of O-H, 2933 cm^-1^ was the stretching vibration peak of C-H such as sugars CH_3_, CH_2_ and CH; the absorption peak of 1700-1500 cm^-1^ was caused by symmetric and asymmetric stretching vibration of C = O; the absorption peak of 1746 cm-1 indicated that the polysaccharide might have uronic acid structure; 1 650-1 500 cm^-1^ was the stretching vibration peak of C-H There was a strong absorption peak at 550 cm^-1^, which was the stretching vibration of C = O and the asymmetric stretching vibration of C = O. The polysaccharide may contain - COOH, indicating that it contains acidic polysaccharide; *Schisandra chinensis* polysaccharide has 834 cm^-1^ and 892 cm^-1^ characteristic absorption peaks, indicating that it may contain α - and β - configuration polysaccharides. The absorption peak of 1200-850 cm^-1^ indicated that the polysaccharide may contain proteoglycan.


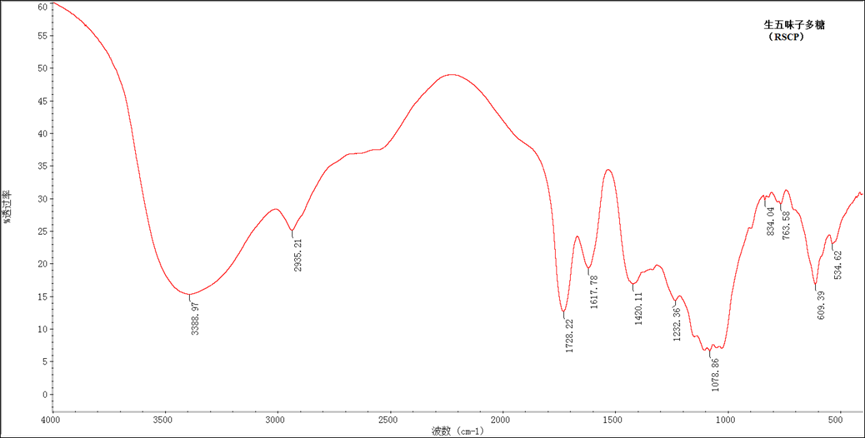


**Figure 2** Infrared spectrum analysis of *Schisandra chinensis* polysaccharide

**2. Western blot analyses**

Colon tissues (100 mg each) were homogenized in ice-cold lysis buffer (RIPA, 1 mM PMSF, PMSF/RIPA=1/100). Homogenates were centrifuged at 12,000 × g for 15 min at 4 °C. The supernatants were collected and centrifuged again, and the final supernatants were collected for the detection of IL-1β, IL-10, IL-23,TNF-α and IFN-γ. Nuclear and cytoplasmic extracts for western blotting were obtained by using a nuclear/cytoplasmic isolation kit (Solarbio® Biotechnology, Beijing, China). Protein levels were determined using the BCA Protein Assay Kit (Thermo Fisher, 23227). Samples (50 μg each) were separated by denaturing SDS-PAGE and collected on a PVDF membrane (0.45 μm, Merck Millipore, USA) by electrophoretic transfer (Mini-Protean® 3 Cell, Bio-Rad, USA). The membrane was pre-blocked with 5% BSA and 0.1% Tween-20 in Tris-buffered saline (TBST) and incubated overnight with the primary antibody (in TBST with 5% BSA). Each membrane was washed three times for 30 min and incubated with the secondary horseradish peroxidase-linked antibodies (Affbiotech, USA). Quantitation of detected bands was performed with the ImageQuantTM TL analysis software (General Electric, USA). To correlate protein loading, the blots were analyzed for β-actin expression using an anti-β-actin antibody (Proteintech Group, Inc，20536-1-AP，1：10000). Each density was normalized using each corresponding β-actin density as an internal control and averaged from three samples. The density of the control was set at 1.0 to compare other groups.

**3. Microbial diversity analysis**

*3.1 DNA extraction and PCR amplification*

Microbial DNA was extracted from 34 samples using the E.Z.N.A.® soil DNA Kit (Omega Bio-tek, Norcross, GA, U.S.) according to the manufacturer’s protocols. The final DNA concentration and purification were determined by NanoDrop 2000 UV-vis spectrophotometer (Thermo Scientific, Wilmington, USA), and DNA quality was checked by 1% agarose gel electrophoresis. The V3-V4 hypervariable regions of the bacteria 16S rRNA gene were amplified with primers 338F (5’- ACTCCTACGGGAGGCAGCAG-3’) and 806R (5’-GGACTACHVGGGTWTCTAAT-3’) by thermocycler PCR system (GeneAmp 9700, ABI, USA). The PCR reactions were conducted using the following program: 3 min of denaturation at 95 °C, 27 cycles of 30 s at 95 °C, 30s for annealing at 55 °C, and 45s for elongation at 72 °C, and a final extension at 72 °C for 10 min. PCR reactions were performed in triplicate 20 μL mixture containing 4 μL of 5 × FastPfu Buffer, 2 μL of 2.5 mM dNTPs, 0.8 μL of each primer (5 μM), 0.4 μL of FastPfu Polymerase and 10 ng of template DNA. The resulted PCR products were extracted from a 2% agarose gel and further purified using the AxyPrep DNA Gel Extraction Kit (Axygen Biosciences, Union City, CA, USA) and quantified using QuantiFluor™-ST (Promega, USA) according to the manufacturer’s protocol.

*3.2 Illumina MiSeq sequencing*

Purified amplicons were pooled in equimolar and paired-end sequenced (2 × 300) on an Illumina MiSeq platform (Illumina, San Diego, USA) according to the standard protocols by Majorbio Bio-Pharm Technology Co. Ltd. (Shanghai, China).

*3.3 Processing of sequencing data*

Raw fastq files were quality-filtered by Trimmomatic and merged by FLASH with the following criteria: (i) The reads were truncated at any site receiving an average quality score <20 over a 50 bp sliding window. (ii) Sequences whose overlap being longer than 10 bp were merged according to their overlap with mismatch no more than 2 bp. (iii) Sequences of each sample were separated according to barcodes (exactly matching) and Primers (allowing 2 nucleotide mismatching), and reads containing ambiguous bases were removed.

Operational taxonomic units (OTUs) were clustered with 97% similarity cutoff using UPARSE (version 7.1 http://drive5.com/uparse/) with a novel ‘greedy’ algorithm that performs chimera filtering and OTU clustering simultaneously. The taxonomy of each 16S rRNA gene sequence was analyzed by the RDP Classifier algorithm (http://rdp.cme.msu.edu/) against the Silva (SSU123) 16S rRNA database using the confidence threshold of 70%.

**4. Representative total ion flow diagram for GC-MS quantitative analysis of SCFAs**


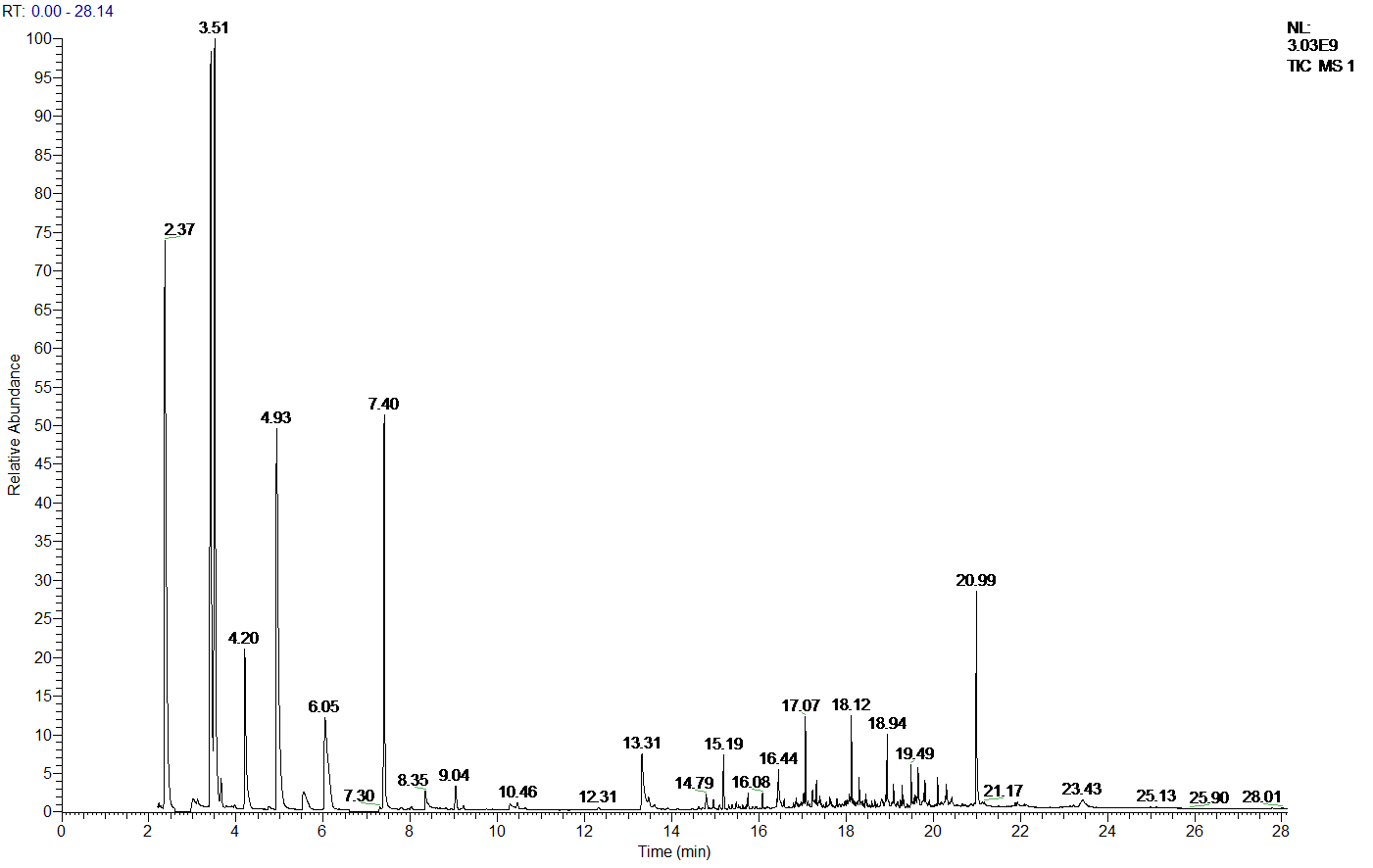


**Figure 3** Total ion current diagram of mixed standard of six kinds of SCFAs


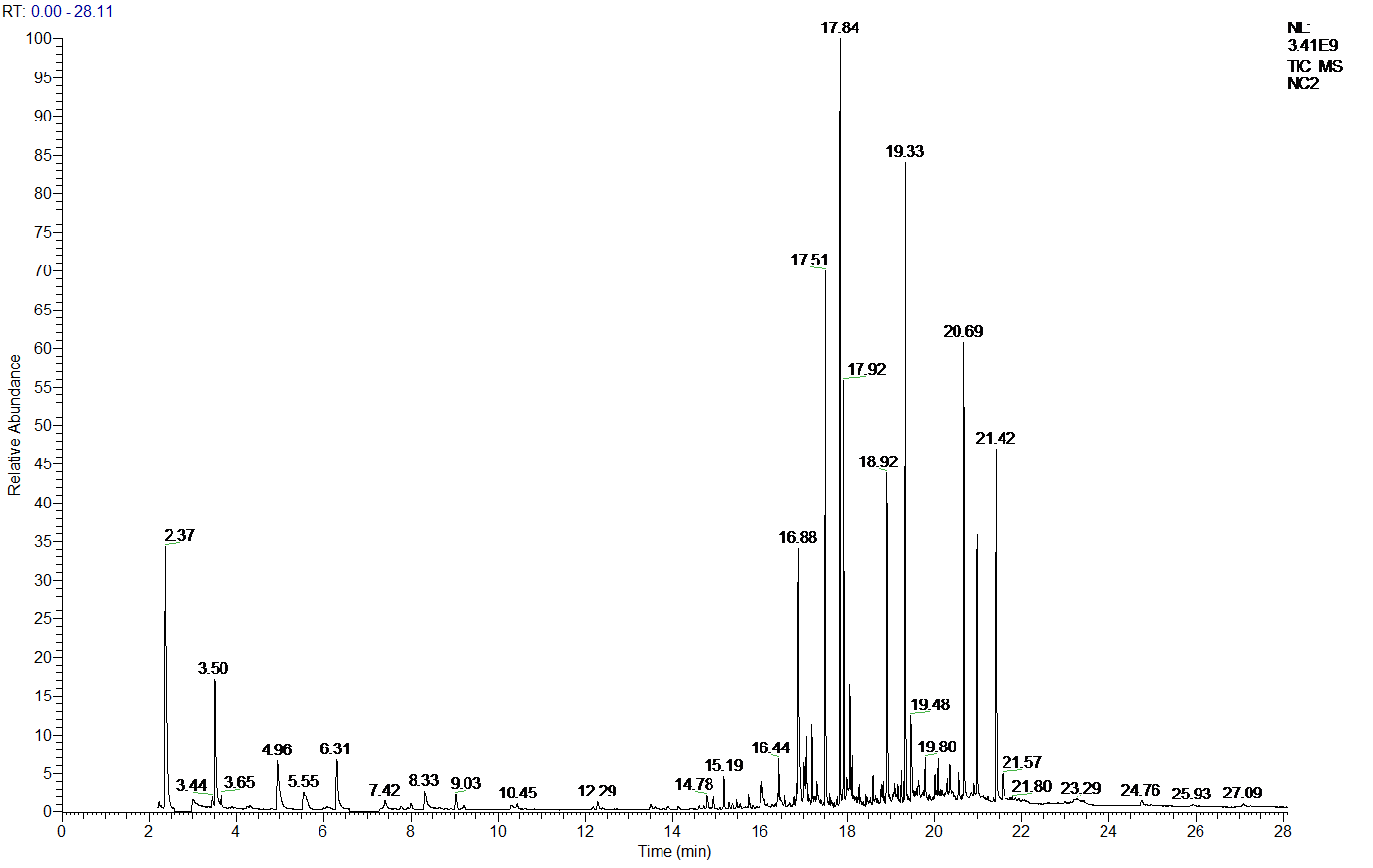


**Figure 4** Total ion current diagram of mixed standard of NC group


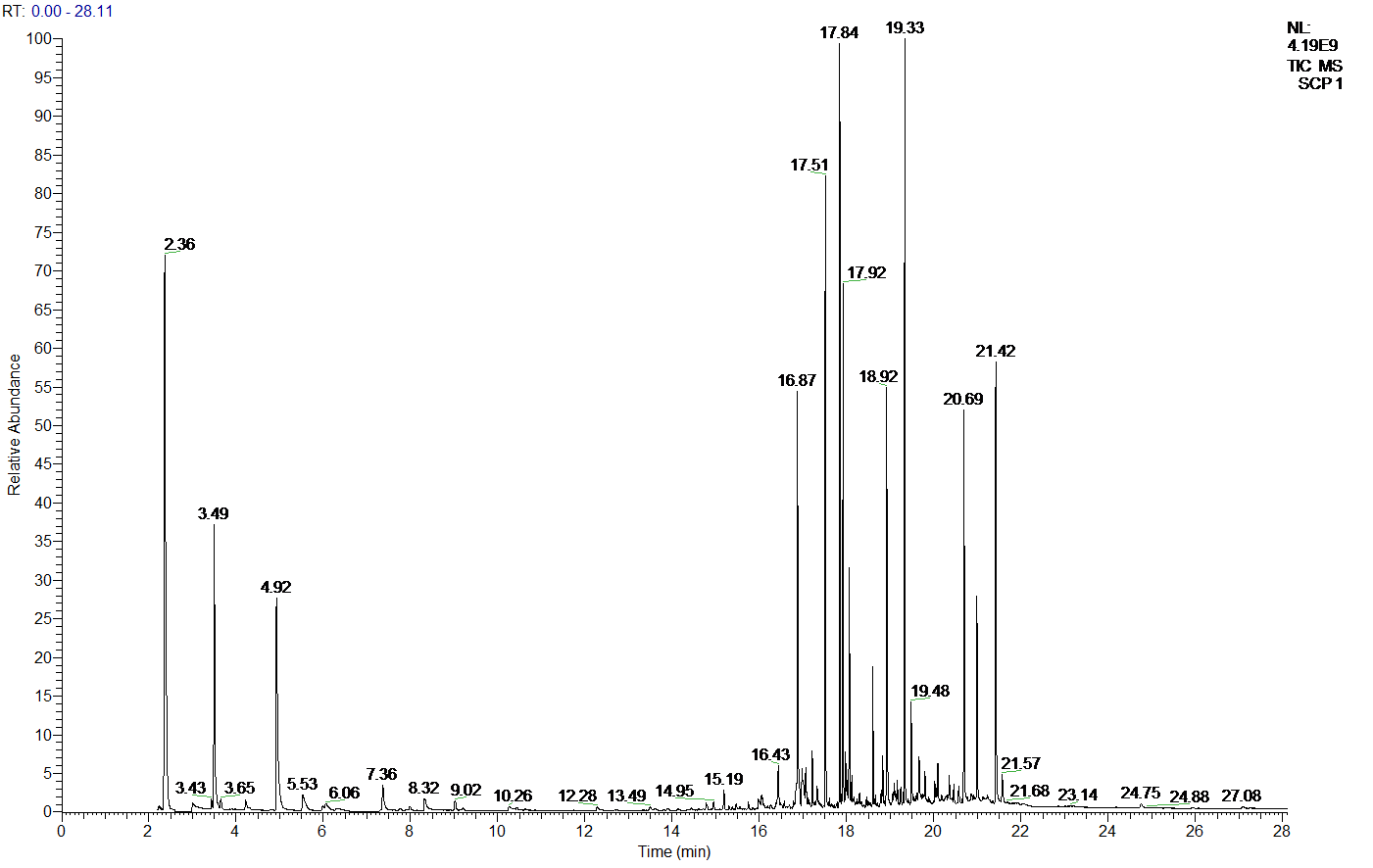


**Figure 5** Total ion current diagram of mixed standard of SCP group
